# O2.1. FIRST EPISODE PSYCHOSIS PATIENTS ACROSS EUROPE DIFFER IN INTELLECTUAL QUOTIENT (IQ) AND EXPOSURE TO ENVIRONMENTAL HAZARDS

**DOI:** 10.1093/schbul/sby015.191

**Published:** 2018-04-01

**Authors:** Laura Ferraro, Marta Di Forti, Caterina La Cascia, Giada Tripoli, Lucia Sideli, Ilaria Tarricone, Diego Quattrone, Robin Murray, Daniele La Barbera

**Affiliations:** 1 University of Palermo; 2 Institute of Psychiatry, Psychology & Neuroscience, King’s College London; 3 Bologna University

## Abstract

**Background:**

Children who later develop Schizophrenia on average are more likely to present with lower IQ; this has been considered evidence for the neurodevelopmental theory of schizophrenia. Though, recent studies have shown that first episode psychosis patients with a history of cannabis use have significantly higher premorbid and current IQ compared to those who never used it. This suggests that abnormal early neurodevelopment does not explain the aetiology of all cases of Schizophrenia, leaving space to environmental hazards.

The present study aims to: investigate differences in IQ, as a marker of neurodevelopment, and in exposure to environmental risk factors in a large sample first episode psychosis patients recruited across five different European countries, in comparison with their respective control groups.

**Methods:**

We analysed data on IQ, socio-demographics and cannabis use from FEP=705 (51.1 % males) and healthy controls=1.034 (48.9 % males), as part of the European network of national schizophrenia networks studying European Gene-Environment-Interaction (EUGEI) study. Patients met ICD-10 criteria for psychosis, ascertained by using OPCRIT (McGuffin et al., 1991).

The CEQmv(Di Forti et al., 2009) further modified for the EUGEI study, was used to collect data on cannabis use. We used ANOVAs where IQ was used as the outcome variable and case/control status and Country were respectively entered as independent predictors, along with other predictors.

**Results:**

Case-control status (F (1,1.484)=133.1, p<0.001) and Country (F (4, 1.484)=32.1, p<0.001) resulted in interaction in predicting IQ after controlling for gender, age, ethnicity, education, occupation relationship and living status. That means, being a case and being from France (mean IQ=73.4), Spain (mean IQ=74.6) and Italy (mean IQ=75.4) was associated with the lowest IQ (F (4,1.484)=3.7, p=0.004), compared with cases from UK (mean IQ=87.1) and Holland (mean IQ=85.5). Among controls the pattern was similar but not significant.

We then grouped countries as North- (UK and Holland) and South – Europe (Italy, Spain, France) and we compared the presence of the main risk factors between the two groups. Both patients and controls from the northern part of Europe, were more likely to be from other ethnicities (chi2(2)=93.3, p<0.001) and living alone (chi2(2)=39.6, p<0.001) than patients and controls from the southern part, who were, for instance, more likely to be married (chi2(2)=34.1, p=0.007). There were no differences in education, nor in gender distribution between cases from the north and from the south of Europe and cases from the south, but not controls, were more likely to be employed than patients from the North (chi2(1)=19.1, p<0.001). A significant difference emerged in patterns of cannabis use, that resulted more dangerous in the northern part, where cases, but not controls, were more likely to have used cannabis (chi2 (1)=18.2, p<0.001) on a daily basis (chi2(2)=17.6, p<0.001) with a higher concentration in THC (chi2 (2)=43.8, p<0.001) and before their 15 years (chi2 (2)=20.8, p<0.001), compared to patients from the south.

**Discussion:**

Our findings on the higher IQ reported in the first episode psychosis patients from northern Europe sites might indicate this as a group with less neurodevelopmental abnormalities and more likely to have developed Psychosis because of adverse social environment and more harmful pattern of cannabis use, compared to patients from the southern countries.

